# Twin-Wire Networks for Zero Interconnect, High-Density 4-Wire Electrical Characterizations of Materials

**DOI:** 10.34133/2022/9874249

**Published:** 2022-01-11

**Authors:** Nerio Andrés Montoya, Valeria Criscuolo, Andrea Lo Presti, Raffaele Vecchione, Christian Falconi

**Affiliations:** ^1^Department of Electronic Engineering, University of Rome Tor Vergata, Via del Politecnico 1, Roma 00133, Italy; ^2^School of Physics, Universidad Nacional de Colombia, A.A. 3840 Medellín, Colombia; ^3^Center for Advanced Biomaterial for Health Care, Istituto Italiano di Tecnologia, Largo Barsanti e Matteucci 53, Naples 80125, Italy

## Abstract

Four-wire measurements have been introduced by Lord Kelvin in 1861 and have since become the standard technique for characterizing small resistances and impedances. However, high-density 4-wire measurements are generally complex, time-consuming, and inefficient because of constraints on interconnects, pads, external wires, and mechanical contacts, thus reducing reproducibility, statistical significance, and throughput. Here, we introduce, systematically design, analyze, and experimentally validate zero interconnect networks interfaced to external instrumentation by couples of twin wire. 3D-printed holders with magnets, interconnects, nonadhesive layers, and spacers can effortlessly establish excellent electrical connections with tunable or minimum contact forces and enable accurate measurements even for delicate devices, such as thin metals on soft polymers. As an example, we measured all the resistances of a twin-wire 29-resistor network made of silver-nanoparticle ink printed on polyimide, paper, or photo paper, including during sintering or temperature calibration, resulting in an unprecedentedly easy and accurate characterization of both resistivity and its temperature coefficient. The theoretical framework and experimental strategies reported here represent a breakthrough toward zero interconnect, simple, and efficient high-density 4-wire characterizations, can be generalized to other 4-wire measurements (impedances, sensors) and can open the way to more statistically meaningful and reproducible analyses of materials, high-throughput measurements, and minimally invasive characterizations of biomaterials.

## 1. Introduction

The electrical characterization of small resistances and impedances almost always requires 4-wire measurements in order to avoid large errors due to series parasitics. This is, for instance, generally the case for conducting materials (*e.g.*, metals) which are widely used in interconnections, electrodes [1], sensors (*e.g.*, temperature [2] or strain sensors [3]), actuators [4], MEMS/NEMS [5, 6], batteries and supercapacitors [7], seed layers [8], wearable [2, 9], or implantable [5, 6] devices and more. However, with conventional 4-terminal measurements, the characterization of *N*_*R*_ integrated [10] devices requires that the numbers of interconnects (*N*_*I*_), pads (*N*_*P*_), and external wires (*N*_*W*_) are all equal to 4*N*_*R*_, which can be an issue for high-density measurements. With reference to the numbers of pads and wires, for high-volume production, permanent connections to pads with very small lateral dimensions may be implemented by ACF (anisotropic conductive film) cables [2, 11–13] or hybrid bonding (with pitches down to few *μ*m), but for preliminary tests or if connections must be established and released or if there are process compatibility issues (*e.g.*, devices may not withstand the temperatures required for bonding), the number of pads can be severely restricted. In fact, mechanical probe stations, beside complexity, cost, and large dimensions, generally have very few test points [14]. Macroscopic connectors (*e.g.*, spring-loaded pins) typically require contact pads with lateral dimensions in the millimeter range [9, 15–20], thus limiting the number of pads, and can easily damage delicate devices (*e.g.*, thin metals deposited on elastic polymers such as PDMS [3, 15, 16, 21]). The number of wires and pads can be reduced by sharing the ground terminal or by simplifying structures used for characterizing mismatch [22], but these approaches still require interconnects, which are invasive (*i.e.*, conductors connecting to the materials under test must be added), increase area and opacity (in case of transparent substrates and opaque conductors), can prevent full characterization of the materials under test (interconnects are not measurable), waste materials (in case of additive manufacturing) with related issues (environmental, economic), and require more complex or time-consuming fabrication (in case of serial manufacturing). An array of resistors without interconnects has been fabricated by writing a conductive line on preexisting pads [23], but 28 pads were used for monitoring only 12 resistors and similar resistors, with very small (in comparison with the pad size) distances between adjacent pads, complicate models, limit accuracy, and waste device area. Due to all these hurdles, there is no standard for the electrical characterization of conducting materials, and in most cases, very few resistances are measured, despite typically large spread (even for nominally identical materials, fabricated at the same time and on the same substrate), thus limiting accuracy, statistical significance, and reproducibility. High-throughput methods used for high-density estimations of the resistivity within nonhomogeneous films (as required, for instance, for data-driven material science [24]) could help, but generally require interconnects made of out-of-plane electrical probes such as spring-loaded pins [25–30] which also have several issues, including the invasive nature of spring probes, possible damages (*e.g.*, scratch), inappropriateness for bent (nonflat) films, roughly determined measurement areas (*i.e.*, each measurement is not exactly associated to a certain region), and accuracy limited by the geometrical correction factor *F* [30] (which depends in a complex way on the resistivity distribution within the thin film).

Here, we introduce, design, theoretically, analyze and experimentally validate twin-wire networks for high-density 4-terminal measurements which require no interconnects and few pads and external wires. Afterwards, we show that these networks can be easily applied even in case of delicate devices, such as thin metals on soft polymers, by means of 3D-printed or PCB (printed circuit board) holders with auxiliary magnets, PCB wires, nonadhesive layers, and spacers. Finally, as an example, we characterize all the resistors of zero interconnect twin-wire 29R networks made of silver-conductive-ink printed on polyimide, paper, or photographic paper. Twin-wire networks enable zero interconnect and unprecedentedly simple, efficient, high-density, and complete 4-wire electrical characterizations.

## 2. Results

### 2.1. Twin-Wire Networks: Theory, Systematic Design, and Analysis

**Figure 1:**
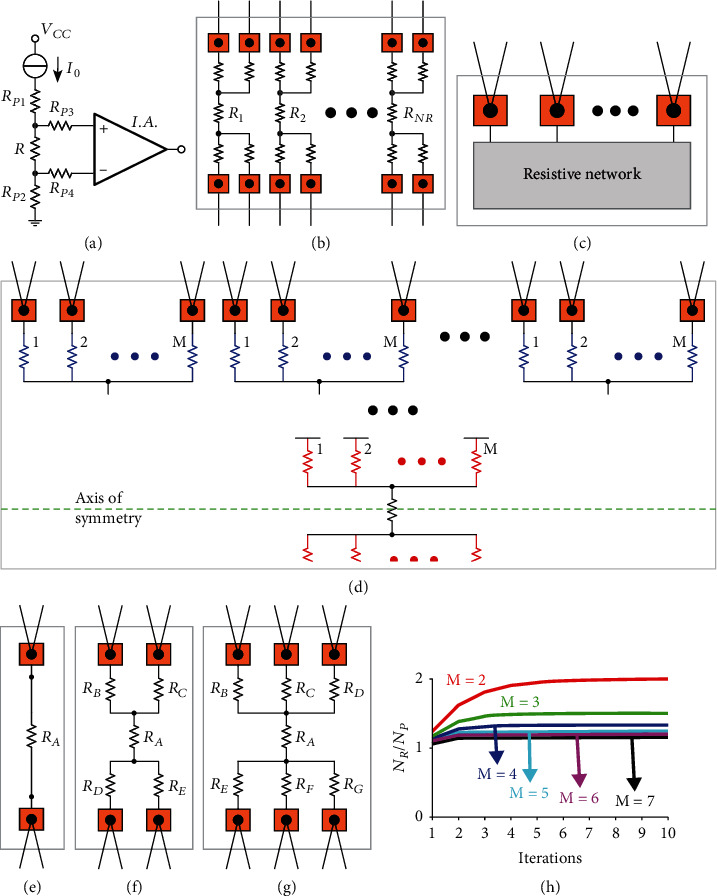
Twin-wire resistive networks. (a) Four-wire measurements. (b) One-wire per pad, 4-wire connections for *N*_*R*_ resistors, with 4*N*_*R*_ pads (orange squares). (c) Schematic representation of twin-wire resistive networks. (d) Iterative multiply-by-*M* expansion procedure for designing twin-wire networks (only half of the symmetric device is shown). (e) Single resistor with 2 pads and twin wires. (f) Five resistors obtained by applying a single bifurcation (*M* = 2) expansion to a single initial resistor. (g) Seven resistors obtained by applying a single trifurcation (*M* = 3) expansion to a single initial resistor. (h) Ratio between the number of resistors, *N*_*R*_, and the number of pads, *N*_*P*_, for multiply-by-*M* expansion procedures as a function of the number of iterations for different values of *M*.

Figure 1(a) schematically shows 4-terminal measurements [31]. Ideally, since the instrumentation amplifier has zero input currents, the entire current *I*_0_ flows through *R*, and therefore, the voltage across *R* is exactly *RI_0_*. Moreover, since *R*_P3_ and *R*_P4_ are in series with the input terminals of the instrumentation amplifiers, their currents and, therefore, their voltages (Ohm's law) are zero, so that the input and the output voltages of the instrumentation amplifier I.A. are exactly equal to *RI_0_* and to *ARI_0_*, respectively, where *A* is the instrumentation amplifier gain. In practice, this approach allows to almost perfectly measure the resistor of interest, *R*, independently on the parasitics *R*_Pk_ and allows to simultaneously measure many resistors. However, as schematically illustrated in Figure 1(b), where pads (orange squares) are explicitly shown, the bijective (one-to-one) correspondence between wires and pads dictates that characterizing *N*_*R*_ integrated resistors requires 4*N*_*R*_ interconnects and (*N*_*R*_, *N*_*P*_, *N*_*W*_) = (*N*_*R*_, 4*N*_*R*_, 4*N*_*R*_), where *N*_*P*_ and *N*_*W*_ are the numbers of pads and external wires, respectively. Since all the grounded force pads can be replaced by one shared grounded pad, 3*N*_*R*_ + 1 wires and, therefore, 3*N*_*R*_ + 1 pads may be sufficient. As shown in Fig. S1, the number of pads and wires may be further reduced to *N*_*R*_ + 1 and *N*_*R*_ + 3, respectively, by connecting all the *N*_*R*_ resistors in series (resulting in *N*_*R*_–1 internal nodes and in 2 external nodes) and introducing one pad, with its interconnect and its external wire, for each internal node and one pad, with two distinct external wires, for each external node (*i.e.*, a simplified version of a structure for characterizing the matching of integrated resistors [22]). However, even with this solution, (*N*_*R*_ + 1) interconnects would still be required. As shown in Figure 1(c), assuming pads can be considered ideal short circuits, in addition to reducing the number of pads and wires, the interconnects can be completely removed without degrading accuracy by connecting each pad with 2 external wires, provided that proper networks are designed. The parasitic resistances of both the wires connecting a pad to instrumentation will typically be small and of similar magnitude, so that even taking into account the nonidealities of the electronic interface, the 2 wires of a pad can be considered interchangeable and will therefore be referred to as twin wires. Figure 1(d) schematically shows an iterative procedure for designing twin-wire networks; for simplicity, taking advantage of the symmetry with respect to the dashed green axis, only half device is shown. In practice, at every step of the iteration, *M* resistors can be added to each free (*i.e.*, not connected to other resistors) terminal of the network and, after the final iteration, all the pads, each with its couple of distinct twin wires, can be added to the free terminals (*i.e.*, to the free terminals of the most peripheral resistors). Figure 1(e) shows a single resistor with (*N*_*R*_, *N*_*P*_, *N*_*W*_) = (1, 2, 4), which corresponds to the simplest twin-wire network (zero iterations). Though, in contrast with conventional 4-terminal measurements (Figure 1(b)), the entire pad-to-pad resistance is measured, this is often not an issue because of the typically very small pad parasitic resistances. Figure 1(f) shows a device with (*N*_*R*_, *N*_*P*_, *N*_*W*_) = (5, 4, 8) obtained by applying to an initial resistor a single bifurcation (*M* = 2) expansion (the cases of 2 and 3 iterations are shown in Fig. S2). Figure 1(g) shows a device with (*N*_*R*_, *N*_*P*_, *N*_*W*_) = (7, 6, 12) obtained by applying to an initial resistor a trifurcation (*M* = 3) expansion (the case of 2 iterations is shown in Fig. S3). In general, for multiply-by-*M* expansions (*i.e.*, *M* = 2 for bifurcation, *M* = 3 for trifurcation, and *M* = 4 for quadrifurcation) with a single initial resistor, the final device will have

(1) $$\begin{array}{c} \left(N_{R},N_{P},N_{W}\right)=\left(1+2\overset{S}{\underset{k=1}{\sum }}M^{k},2M^{S},4M^{S}\right)=\left(1+2\left[\frac{M^{S+1}-M}{M-1}\right],2M^{S},4M^{S}\right), \end{array}$$

where *S* is the number of recursive steps or iterations (Note S1). The possibility to accurately measure many resistors with a small number of pads can be quantified by

(2) $$\begin{array}{c} \frac{N_{R}}{N_{P}}=\frac{1+2\left[\left(M^{S+1}-M\right)/\left(M-1\right)\right]}{2M^{S}}, \end{array}$$

which, as shown in Figure 1(h), when the number of iterations is increased, quickly tends to

(3) $$\begin{array}{c} \underset{S⟶\infty }{\lim}\frac{N_{R}}{N_{P}}=\frac{M}{M-1} \end{array}$$

(Note S1). Though each design problem should be separately analyzed (*e.g.*, for *N*_*R*_ = 7 the circuit shown in Figure 1(g) may be more effective), *N*_*R*_/*N*_*P*_ decreases for increasing *M* (*N*_*R*_/*N*_*P*_ tends to values approaching 1 for increasing *M*) and is maximum for bifurcation. However, different choices may have other advantages. For instance, trifurcation is more robust than bifurcation against accidental open circuits because if a single resistor is open-circuited, all the residual resistors can still be individually measured (Note S2, Fig. S4). More in general, there can be an arbitrary number of initial resistors, and at every iterative step, the number of resistors added to a certain free terminal can be zero or any natural number equal to or larger than 2 (Fig. S5) because adding a single resistor would result in two series (*i.e.*, indistinguishable) resistors. The network automatically implements all the internal wires without the need for any additional interconnect. Though different twin-wire networks can be designed, with these strategies, every resistor can be connected to instrumentation by two force pads and two sense pads and can be individually determined with a single measurement (Fig. S6).

### 2.2. Tunable-Force Magnetic Connectors for Twin-Wire Networks

**Figure 2:**
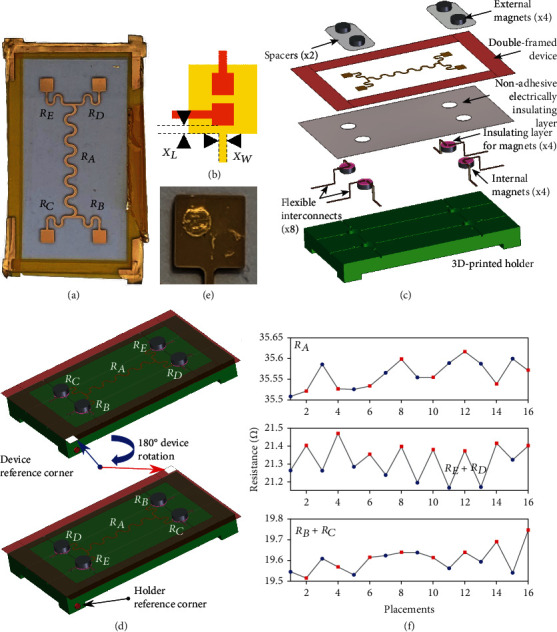
Twin-wire thin gold resistors on PDMS and tunable-force magnetic connectors. (a) Zero Interconnect 5-resistor device with only 4 pads. (b) Schematic model for estimating the order of magnitude of parasitic pad resistances. (c) Schematic representation (exploded view) of the measurement system, consisting in (bottom to top) 3D-printed holder, internal magnets, nonadhesive electrically insulating layer for internal magnets, flexible interconnects, nonadhesive electrically insulating layer, double-framed device, spacers, and external magnets (please, see Fig. S7 for details). (d) Schematic representation of the validation experiment consisting in repeatedly placing on the holder, characterizing, removing, rotating (180°), and then placing back on the holder the device, with the holder reference corner coincident (top) or opposite (bottom) with respect to the device reference corner. (e) Photos of typical signs left on the thin gold pads by flexible PCB interconnects with circular electrodes after tens of repeated connections and disconnections. (f) Resistances *R*_*A*_, *R*_*E*_ + *R*_*D*_, and *R*_*B*_ + *R*_*C*_ measured in 16 consecutive experiments with the holder reference corner coincident (odd experiment numbers, blue data points) or opposite (even experiment numbers, red data points) with respect to the device reference corner.

As a remarkable advantage, twin-wire networks do not need any interconnect and external instrumentation must only be connected to the most peripheral elements. However, it is still crucial to guarantee that very good electrical contacts can be established without damaging the device as, unlike the case of conventional 4-wire resistors (Figure 1(b)), the parasitic pad resistances *R*_PAD_ will affect measurements and must therefore be minimized. This problem can be critical for devices where conductors (*e.g.*, thin metals on soft polymers) are so delicate that *R*_PAD_ may be significantly increased even when connecting with instrumentation. For validation, we fabricated [32] zero interconnect twin-wire gold resistors on PDMS (Figure 2(a)). In practice, 10 nm chromium adhesion layer and 100 nm gold were thermally evaporated on 50 *μ*m thick PDMS. The devices were double-framed [32] for simplifying both fabrication and handling. As schematically shown in Figure 2(b), the order of magnitude of the worst case parasitic pad resistance *R*_PAD,WC_ can be estimated by considering a hypothetical resistor with length, width, and thickness equal to *X*_*L*_, *X*_*W*_, and *t*, respectively, so that

(4) $$\begin{array}{c} R_{\text{PAD},\text{WC}}=\rho_{m}\frac{X_{L}}{{tX}_{W}}, \end{array}$$

where *ρ*_*m*_ is the metal electrical resistivity. With conventional 4-terminal sensing (Figure 1(b)), *R*_PAD_ would be excluded from the measurements and only exceptional increases (*e.g.*, open circuit or extreme values which do not allow proper operations of the current source) would be problematic. By contrast, with twin-wire connections, though *R*_PAD_ can be negligible (*e.g.*, in the order of 0.2 *Ω* for our devices, Note S3), significant increases due to damage could degrade accuracy. The measurement system schematically shown in Figure 2(c) (exploded view) can easily guarantee the substantial integrity of the pads even after repeated connections and disconnections. In practice, a 3D-printed holder with cavities, in correspondence of the pads, for hosting magnets enables gentle connections after electrically insulating nonadhesive layers, flexible twin-wire PCB interconnects, the device and spacers are placed between the internal and the external magnets. The magnetic forces can obviously be tuned by selecting proper magnets and spacers. The contact force applied by the magnets can, in principle, be measured (for instance by taking advantage of a spacer comprising a load cell), but in most cases, even for delicate devices, a simple estimation can be sufficient. For instance, for conventional cylindrical magnets such as the ones used in our experiments (S-08-03-E, 8 mm diameter, 3 mm height and 14.7 N attractive force in absence of spacers, N45 magnetisation), the force may be estimated to range from a theoretical maximum of 14.7 N in absence of any gap to about 2.45 N for a total distance between the magnets equal to 3 mm. If the devices have sufficiently thick substrates, spacers can be omitted. Details on fabrication are shown in Fig. S7. For validation, we repeatedly placed on the holder, removed, rotated (180°), and placed back on the holder the device, with the holder reference corner coincident (top) or opposite (bottom) with respect to the device reference corner (Figure 2(d)). Tens of repeated connections and disconnections only left slight marks on the pads (Figure 2(e)) but, beside not giving any loss of electrical continuity or functional problem, only resulted in very small changes of both (*R*_*B*_ + *R*_*C*_) and (*R*_*D*_ + *R*_*E*_), thus confirming negligible increases of *R*_PAD_ (Figure 2(f), Note S4). If necessary, softer interconnects and different magnets or spacers can be adopted for establishing even more delicate connections. For minimum-force contacts, the thickness of spacers can be gradually reduced. The same strategies can be applied to PCB holders having magnets attached on the back side and counter pads in correspondence of the device pads (Fig. S8).

### 2.3. Twin-Wire 29R Networks: Electronic Interface and Experiments

**Figure 3:**
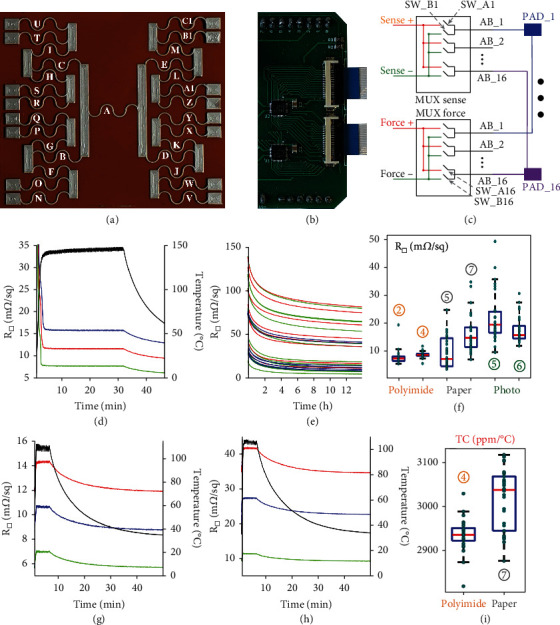
Twin-wire 29R networks: electronic interface and experiments. (a) Twin-wire 29R network made of silver-nanoparticles conductive ink printed on polyimide. Part of the control PCB interface (b) for connecting the twin-wire 29R to sense or force terminals by switches (c). (d) Mean value, minimum, and maximum sheet resistances for a device printed on polyimide and temperature (black signal) during sintering. (e) Sheet resistances for the 29 resistors of a device printed on photo paper during room-temperature sintering. (f) Box-and-whisker plot illustrating the spread of the room temperature sheet resistances for 6 representative devices printed on polyimide, paper, or photo paper. Mean value, minimum and maximum sheet resistances for two devices printed on polyimide (g) or paper (h) during a temperature calibration experiment. (i) Box-and-whisker plot illustrating the spread of the temperature coefficient of the sheet resistances for 2 devices printed on polyimide and paper.

Twin-wire networks can enable zero interconnect and unprecedentedly simple, effective, and massive 4-wire characterization of conductors, which is often crucial for material science. As an especially relevant example, the electrical resistivity of conductive inks is widely investigated, including its dependence on ink formulation [33, 34], nanoparticle diameter [35], substrates [36], sintering temperature [34] and duration [36, 37], dot spacing [38], trace width [39], strain/bending [33, 39, 40], number of strain/bending cycles [40], and strain rate [39]. In all these cases, twin-wire networks can provide key advantages. As a proof of concept, as shown in Figure 3(a), we printed zero interconnect twin-wire 29R networks made of silver nanoparticles conductive ink on different substrates, namely, a 75 *μ*m thick polyimide substrate, conventional paper, and Epson Premium Glossy photographic paper. With these relatively robust devices, the electrical connections can be simply established by attaching to the bottom side of the substrate small pieces of metals and by inserting a previously printed polyimide connector between the device and top magnets. The inclusion of internal pads is not required but simplifies the direct access with probes to each resistor for preliminary tests or additional measurements. If a part of an internal pad belongs to both the force and the sense paths, its resistance must be taken into account when estimating the sheet resistance (*e.g.*, corner resistors such as *R*_*N*_), while in other cases, it is automatically excluded by 4-wire measurements (*e.g.*, central resistor *R*_*A*_). Figure 3(b) shows a part of the PCB control board comprising the twin wires with an array of switches which, as schematically shown in Figure 3(c), can connect the pads to the force or sense terminals of a conventional multimeter. The PCB control board is an Arduino shield connected to the multimeter by a BNC cable for synchronization (Fig. S9, Note S5). As a first test, immediately after printing the device on a 75 *μ*m thick polyimide substrate, we monitored the 29 resistors during sintering induced by increasing the temperature of the plate supporting the device. Similar to a previous report [41] on real-time sintering monitoring, the pads (but not the interconnects which, in our case, are not required) were printed and sintered before printing the network. Though the magnets simplify the connection and the pad-connector alignment, the temperature required for sintering (though much lower than the Curie temperature) may reduce their strength, so, to avoid any issues, we placed on top of the magnets a rigid PCB as a support for some weight to keep the connector in position even under reduced magnetic forces. Figure 3(d) shows the temperature and 3 representative sheet resistances during sintering (mean value, minimum, and maximum; the 29 sheet resistances are shown in Fig. S10). We also measured the 29 sheet resistances of a device printed on Epson photo paper during room-temperature sintering triggered by [poly(diallyldimethylammonium chloride]-like molecules [42] (Figure 3(e)), thus revealing that even after 14 hours the resistances are still slowly decreasing and large mismatch in the sintering dynamics. Figure 3(f) shows the box-and-whisker plot for the 29 room temperature resistances of 6 representative devices printed on polyimide, paper, and photo paper. We also fabricated and characterized other devices printed on polyimide and photo paper (Fig. S11) or on paper (Fig. S12). Since paper can already be altered by the recommended ink sintering procedure (around 145°C, measured by the Pt100 sensor, for 45 minutes), we also printed devices which were not heated for sintering (paper device 2) or kept at 100°C for 1 hour (paper device 3). As expected, the device kept at room temperature exhibited the highest mean sheet resistance, but all resistors worked properly. Interestingly, the paper device 6, though sintered as recommended, had one resistor with sheet resistance above 140 m*Ω*/□, which is much higher than any other resistor in the paper devices. Finally, two devices printed on polyimide and on paper, respectively, were heated up to 100°C by placing them on a temperature-controlled plate which was then switched off, while measuring the 29 resistances and the plate temperature with a Pt100 (Figures 3(g) and 3(h) and Fig. S13). Figure 3(i) shows the box-and-whisker plot for the temperature coefficients *α*(*T*_REF_), with *T*_REF_ equal to 27°C, defined as

(5) $$\begin{array}{c} \alpha \left(T_{\text{REF}}\right)=\frac{1}{R\left(T_{\text{REF}}\right)}\frac{\partial R\left(T_{\text{REF}}\right)}{\partial T}. \end{array}$$

Clearly, in order to make the results independent on the device layout, all these measurements have been expressed in terms of sheet resistance. However, as an example, Figure S14 and Table S1 show the 29 resistances of device 4 printed on polyimide (the same measurements shown in Figure 3(f) and S11), with values of a few hundreds of mΩ which can, for instance, be easily read by a commercial multimeter (Figure S9) with errors, in that resistance range, around ±3 mΩ. However, in general, for wearable or implantable systems, if resistances are very small, as in our case, or very large, special care is required for designing high-accuracy electronic interfaces.

The strategies described here are general and can, for instance, be applied to flexible devices with resistors under bending, folding or any deformation as long as the resistances are not damaged and can be measured. As just a possible example, Figure S15 shows that, after a 29-resistor twin-wire network has been printed on a flexible polyimide substrate, the resistances can be read without any issue even after the device has been bent.

## 3. Discussion

We introduced zero interconnect twin-wire networks for enabling the accurate 4-wire measurement of many impedances or resistors without any interconnects and with reduced numbers of pads and external wires. Iterative multiply-by-*M* procedures allow to easily generate twin-wire networks where each resistor can be individually determined with a single measurement. Bifurcation (*M* = 2) expansions are more effective in terms of the ratio between the numbers of measurable resistors and pads. Other options may have different advantages, and for instance, trifurcation is more robust than bifurcation against accidental open circuits.

With twin-wire networks, the parasitic pad resistances, which are included when measuring the most peripheral resistors, must be kept at a sufficiently low level. In case of delicate devices and of repeated connections and disconnections, 3D-printed or PCB holders with auxiliary magnets, flexible interconnects, nonadhesive layers, and spacers can establish excellent electrical connections with tunable contact forces, thus allowing to safely characterize even delicate devices, such as thin metals on soft elastomers (*e.g.*, PDMS).

For validation, we printed several twin-wire 29-resistor networks made of silver conductive ink on polyimide, paper, or photo paper. For each device, all the 29 resistors could be characterized, with a single experiment, both individually and statistically, by a control board connected with only 16 pads (32 wires) and interfaced to a conventional multimeter. All the 29 printed conductors were characterized during high-temperature (polyimide) or room-temperature (Epson photo paper) sintering. Moreover, the temperature dependences of all the 29 resistances of selected devices were evaluated. Such unprecedentedly accurate analyses on a commercial silver-nanoparticles conductive ink (in terms of sintering, resistivity, temperature coefficient, and substrate-dependence) are just an example as twin-wire networks can be applied to other problems (ink formulation, substrates, sintering methods, strain/bending, and corrosion) or materials.

Twin-wire networks easily allow zero interconnect, complete (the entire material can be characterized as there is no need for additional interconnects) as well as more accurate, statistically meaningful, and reproducible electrical characterizations and can therefore greatly facilitate the standardization of measurements. For instance, our experiments show large spread among nominally identical resistors (the same materials and the same fabrication procedure). Clearly, ignoring such spread would have degraded accuracy, statistical significance, and reproducibility.

The absence of interconnects can result in noninvasive measurements (only the most peripheral elements must be contacted), full characterization of the entire conducting materials, smaller area, superior transparence (in case of opaque conductors and transparent substrates), no material waste, and faster fabrication (in case of additive manufacturing).

If the thin film can be properly patterned (*e.g.*, shadow masks, etching, or lift-off) for creating the resistive network, twin-wire networks can also be used for high-throughput characterizations (required, for instance, for data-driven material science [24]) without interconnects made of out-of-plane electrical probes, thus providing important advantages, including noninvasive measurements, no mechanical damages (*e.g.* scratch), appropriateness for bent (nonflat) films, exactly determined measurement areas, and having no need for complex geometrical corrections.

Twin-wire networks can be included in wearable or implantable flexible systems, no matter the type of resistive elements, including, for instance, nanowire-based [43], 3D-printed [44], or cellulose-based [45] resistors. In principle, such wearable or implantable systems can also be powered by mechanical energy converted into electrical energy by nanogenerators [46–48]. The twin-wire network and its electronic interface can be integrated in a single chip [31], but even in this case, the zero interconnect property can be important for simplifying layout and for minimizing the wires which must enter the network region.

Twin-wire networks can be generalized to impedances, instead of resistances, and can also find additional applications (*e.g.*, twin-wire networks of sensors for high-density measurements with reduced numbers of interconnects, wires, and pads, which is especially important for wearable or epidermal devices).

The zero interconnect property enables minimally invasive characterizations of a multitude of elements with only a few contacts made to the most peripheral elements, which can, for instance, reveal a key advantage for high-throughput measurements (without invasive contacts with the associated risks of damaging the material under test) and noninvasive, high-density characterization of biomaterials.

## 4. Materials and Methods

### 4.1. Fabrication of Thin Gold on PDMS Test Devices

Double-framed thin PDMS devices were fabricated using a 60 *μ*m thick polyimide tape as a first frame on a PMMA substrate. PMMA substrates and shadow masks were micromilled with a Minitech MiniMill. 50 *μ*m thick PDMS was spin coated on PMMA. 10 nm Cr and 100 nm Au were thermally evaporated over the PDMS. A second paper frame was used for neutralizing the adhesive of the polyimide tape in order to facilitate both peeling and the subsequent handling of the device. Details can be found in [32].

### 4.2. Fabrication of the 3D-Printed Holder

The holder was printed with a custom-made 3D-printer Prusa i3, using an ABS+ filament (1.75 mm diameter from Sunlu Industrial Co.) and 0.2 mm copper nozzle. The 3D system was designed with the software Solid Edge ST8 (Siemens PLM software), and Ultimaker Cura 4.6.2 was used for creating the 3D slides file. The neodymium magnets (S-08-03-E, 8 mm diameter, 3 mm height and 14.7 N attractive force in absence of spacers) were purchased from Supermagnete. Paper and parafilm were used as nonadhesive layers to cover the magnets and the holder, respectively. Flexible PCBs were used as flexible interconnects. No spacers or glass spacers were used when characterizing devices before and after peeling, respectively. The flexible PCB connectors for the 3D-printed holder, the PCB holder, the Pt100 sensing PCB, and the Arduino Shield were designed with EAGLE 9.6.2.

### 4.3. Fabrication of Zero Interconnect Twin Network Made of Silver Conductive Ink on Different Substrates

The 29-resistor network was designed with EAGLE 9.6.2 and printed over polyimide (DuPont™ Kapton® HN, 75 *μ*m), common printer paper (Golden Star® Premium), and photo paper (Epson Premium Glossy) using a Voltera V-One multifunctional circuit printer (Voltera Inc., http://www.voltera.io/) with a commercial silver nanoparticles ink (Flexible 2 Ink, Voltera Inc.) and a plastic nozzle (225 *μ*m inner diameter, Voltera Inc.). The silver ink was kept outside the refrigerator for 15 minutes before starting to print. The sintering (for common paper and polyimide devices) was done with the Voltera V-One printer (hotplate surface), using the Voltera-recommended sintering procedure (nominal temperature of 160°C for 45 minutes) and keeping the devices attached to the surface with polyimide adhesive tape (Tesa®, premium grade polyimide tape 51408). No high-temperature sintering procedure was used for the photo paper devices because their coating effectively triggers room-temperature sintering [42]. On selected devices printed over polyimide, the printing and sintering procedure was divided in a first phase for printing and sintering the connection pads and in a second phase for printing and sintering the network. In both cases, the devices were kept attached to the surface and sintered with the Voltera-recommended sintering procedure (nominal temperature of 160°C for 45 minutes).

### 4.4. Measurements and Data Analysis

Four-wire measurements were taken with a Digital Multimeter Agilent 34410A interfaced with the circuit by a PCB comprising an Arduino microcontroller and arrays of switches (Figures 3(b) and 3(c), Fig. S9). During both sintering and the experiments for determining the temperature coefficient of the sheet resistances, the temperature was measured with a standard Pt-100 sensor, placed near the central resistor (*R*_1_) and over the surface of the device (29 resistors network), connected to a second Digital Multimeter Agilent 34410A in 4-wire temperature-measurement mode. Data analysis was performed with MATLAB (R2020a).

## Data Availability

The data that support the findings of this study are openly available in Mendeley Data at https://data.mendeley.com/datasets/pywhr745ns/1 (doi:10.17632/pywhr745ns.1).

## Supplementary Materials

Supplementary Materials Note S1: theoretical analysis of iterative multiply-by-M expansion procedures. Note S2: consequences of open circuits in twin-wire resistive networks. Note S3: estimation of the pad internal parasitic resistance. Note S4: validation experiments (repeated placement-removal and 180° rotations). Note S5: real-time measurement of the 29R twin-wire network. Figure S1: network of NR resistors with NR+1 pads and NR+3 wires. Figure S2: twin-wire networks generated by bifurcation. Figure S3: twin-wire networks generated by trifurcation. Figure S4: effects of open circuits on twin-wire networks. Figure S5: twin-wire networks generated by arbitrary expansion steps. Figure S6: measurability of each resistor in the twin-wire network. Figure S7: schematic illustration of interconnecting systems for twin-wire networks. Figure S8: PCB holder for the electrical characterization of twin-wire networks. Figure S9: system for real-time measurement of 29 resistances. Figure S10: sheet resistances of the twin-wire 29R silver-conductive ink network printed on polyimide during high-temperature sintering. Figure S11: sheet resistances of the 29 resistors for devices printed on polyimide (left) and on photo paper (right). Figure S12: sheet resistances of the 29 resistors for devices printed on paper. Figure S13: temperature calibration for twin-wire 29R networks of silver conductive ink printed on polyimide and on paper. Figure S14: resistances of a twin-wire 29R network printed on polyimide measured at room temperature. Figure S15: characterization of resistances in a twin-wire network under deformation.
