# Supplementary material for: Twin-Wire Networks for Zero Interconnect, High-Density 4-Wire Electrical Characterizations of Materials
Source: Research (Wash D C). 2022 Jan 11;2022:9874249. doi: 10.34133/2022/9874249 (PMC8771198; doi:10.34133/2022/9874249)
Supplement: Supplementary Materials — Note S1: theoretical analysis of iterative multiply-by-M expansion procedures. Note S2: consequences of open circuits in twin-wire resistive networks. Note S3: estimation of the pad internal parasitic resistance. Note S4: validation experiments (repeated placement-removal and 180° rotations). Note S5: real-time measurement of the 29R twin-wire network. Figure S1: network of NR resistors with NR+1 pads and NR+3 wires. Figure S2: twin-wire networks generated by bifurcation. Figure S3: twin-wire networks generated by trifurcation. Figure S4: effects of open circuits on twin-wire networks. Figure S5: twin-wire networks generated by arbitrary expansion steps. Figure S6: measurability of each resistor in the twin-wire network. Figure S7: schematic illustration of interconnecting systems for twin-wire networks. Figure S8: PCB holder for the electrical characterization of twin-wire networks. Figure S9: system for real-time measurement of 29 resistances. Figure S10: sheet resistances of the twin-wire 29R silver-conductive ink network printed on polyimide during high-temperature sintering. Figure S11: sheet resistances of the 29 resistors for devices printed on polyimide (left) and on photo paper (right). Figure S12: sheet resistances of the 29 resistors for devices printed on paper. Figure S13: temperature calibration for twin-wire 29R networks of silver conductive ink printed on polyimide and on paper. Figure S14: resistances of a twin-wire 29R network printed on polyimide measured at room temperature. Figure S15: characterization of resistances in a twin-wire network under deformation. [file 9874249.f1.pdf]

Supplementary materials

Twin-Wire Networks for Zero-Interconnects, High-Density 4-Wire Electrical Characterizations of Materials

*Nerio Andrés Montoya, Valeria Criscuolo, Andrea Lo Presti, Raffaele Vecchione, Christian Falconi**

Note S1 – Theoretical analysis of iterative multiply-by-M expansion procedures

Note S2 – Consequences of open-circuits in twin-wire resistive networks

Note S3 – Estimation of the pad internal parasitic resistance

Note S4 – Validation experiments (repeated placements-removals and 180° rotations)

Note S5 – Real-time measurement of the 29R twin-wire network

Note S1 – Theoretical analysis of iterative multiply-by-*M* expansion procedures

In case of bifurcation (M = 2), starting with a single initial resistor, the number of additional resistors generated by bifurcation is equal to 4 during the first step and, for the following steps, to twice the number of resistors generated in the previous step, thus resulting in

$$\left(N_R, N_P, N_W\right) = \left(1 + \sum_{k=1}^{S} 2^{k+1}, 2^{S+1}, 2^{S+2}\right) \tag{1}$$

In the more general case of iterative multiply-by-*M* expansion procedures (M = 2, 3, 4, …) the number of resistors generated in a certain iteration step is equal to 2*M* during the first step and, for the following multiply-by-*M* steps, is equal to *M* times the number of resistors generated in the previous step, so that

$$N_R = 1 + 2\sum_{k=1}^{S} M^k \tag{2}$$

Since the sum of the first *N* terms of a geometric series is

$$\sum_{k=0}^{N} M^k = \frac{1 - M^{N+1}}{1 - M} \tag{3}$$

it is convenient to rewrite the number of resistors N_R as

$$N_R = 1+2\sum_{k=1}^{S} M^k = 1+2\left[\left(\sum_{k=0}^{S} M^k\right)-1\right] =$$
$$= 1+2\left[\frac{1-M^{S+1}}{1-M}-1\right] = 1+2\left[\frac{1-M^{S+1}-1+M}{1-M}\right] = \tag{4}$$
$$= 1+2\left[\frac{M^{S+1}-M}{M-1}\right]$$

which is obviously positive ($M > 1$).

Similarly, the number of pads is equal to $2M$ after the first expansion step and, for the other cases, is equal to M times the number of pads generated in the previous step, so that

$$N_P = 2M^S \tag{5}$$

and, therefore (twin-wires),

$$N_W = 4M^S \tag{6}$$

The ability of a device to enable the accurate measurement of many resistors with a small number of pads and, therefore, with a small number of wires (twin-wires), can be quantified by the ratio

$$\frac{N_R}{N_P} = \frac{2N_R}{N_W} = \frac{1+2\left[\frac{M^{S+1}-M}{M-1}\right]}{2M^S} \tag{7}$$

As shown in Figure 1h, this ratio, when the iteration index is increased, quickly tends to

$$\lim_{S\to\infty}\frac{N_R}{N_P}=\lim_{S\to\infty}\frac{1+2\left[\dfrac{M^{S+1}-M}{M-1}\right]}{2M^S}=$$

$$=\lim_{S\to\infty}\frac{2\left[\dfrac{M^{S+1}}{M-1}\right]}{2M^S}= \qquad (8)$$

$$=\lim_{S\to\infty}\frac{M^{S+1}}{M^S\left(M-1\right)}=\frac{M}{M-1}$$

which has a maximum, equal to 2 (*i.e.* N_R approaches N_W or, equivalently, $2N_P$), in the case of the bifurcation ($M = 2$) and tends to 1 for increasing M.

The following tables show, for M ranging between 2 and 7, the $\lim_{S\to\infty}\frac{N_R}{N_P}$, the number of iterations (S ranging between 1 and 10), N_R, N_P , N_W and $\frac{N_R}{N_P}$.

***M* = 2**

$$\lim_{S\to\infty}\frac{N_R}{N_P}=\left.\frac{M}{M-1}\right|_{M=2}=2$$

S	N_R	N_P	N_W	N_R / N_P
0	1	2	4	0.5
1	5	4	8	1.25
2	13	8	16	1.625
3	29	16	32	1.8125
4	61	32	64	1.90625
5	125	64	128	1.953125
6	253	128	256	1.9765625
7	509	256	512	1.98828125
8	1021	512	1024	1.994140625
9	2045	1024	2048	1.997070313
10	4093	2048	4096	1.998535156

***M* = 3**

$$\lim_{S\to\infty}\frac{N_R}{N_P}=\left.\frac{M}{M-1}\right|_{M=3}=\frac{3}{2}=1.5$$

S	N_R	N_P	N_W	N_R / N_P
0	1	2	4	0.5
1	7	6	12	1.166666667
2	25	18	36	1.388888889
3	79	54	108	1.462962963
4	241	162	324	1.487654321
5	727	486	972	1.495884774
6	2185	1458	2916	1.498628258
7	6559	4374	8748	1.499542753
8	19681	13122	26244	1.499847584
9	59047	39366	78732	1.499949195
10	2E+05	1E+05	2E+05	1.499983065

M = 4

$$\lim_{S\to\infty}\frac{N_R}{N_P}=\frac{M}{M-1}\bigg|_{M=4}=\frac{4}{3}=1.\overline{3}$$

S	***N_R***	***N_P***	***N_W***	***N_R / N_P***
0	1	2	4	0.5
1	9	8	16	1.125
2	41	32	64	1.28125
3	169	128	256	1.3203125
4	681	512	1024	1.330078125
5	2729	2048	4096	1.332519531
6	10921	8192	16384	1.333129883
7	43689	32768	65536	1.333282471
8	174761	131072	262144	1.333320618
9	699049	524288	1048576	1.333330154
10	3E+06	2E+06	4194304	1.333332539

M = 5

$$\lim_{S\to\infty}\frac{N_R}{N_P}=\frac{M}{M-1}\bigg|_{M=5}=\frac{5}{4}=1.25$$

S	***N_R***	***N_P***	***N_W***	***N_R / N_P***
0	1	2	4	0.5
1	11	10	20	1.1
2	61	50	100	1.22
3	311	250	500	1.244
4	1561	1250	2500	1.2488
5	7811	6250	12500	1.24976
6	39061	31250	62500	1.249952
7	195311	156250	312500	1.2499904
8	976561	781250	1562500	1.24999808
9	4882811	3906250	7812500	1.249999616
10	2,4E+07	2E+07	3,9E+07	1.249999923

M = 6

$$\lim_{S\to\infty}\frac{N_R}{N_P}=\frac{M}{M-1}\bigg|_{M=6}=\frac{6}{5}=1.2$$

S	*N_R*	*N_P*	*N_W*	*N_R / N_P*
0	1	2	4	0.5
1	13	12	24	1.083333333
2	85	72	144	1.180555556
3	517	432	864	1.196759259
4	3109	2592	5184	1.199459877
5	18661	15552	31104	1.199909979
6	111973	93312	186624	1.199984997
7	671845	559872	1119744	1.199997499
8	4031077	3359232	6718464	1.199999583
9	24186469	20155392	40310784	1.199999931
10	1,45E+08	1,21E+08	2,42E+08	1.199999988

***M* = 7**

$$\lim_{S\to\infty}\frac{N_R}{N_P}=\frac{M}{M-1}\bigg|_{M=7}=\frac{7}{6}=1.1\overline{6}$$

S	*N_R*	*N_P*	*N_W*	*N_R / N_P*
0	1	2	4	0.5
1	15	14	28	1.071428571
2	113	98	196	1.153061224
3	799	686	1372	1.164723032
4	5601	4802	9604	1.166389005
5	39215	33614	67228	1.166627001
6	274513	235298	470596	1.166661
7	1921599	1647086	3294172	1.166665857
8	13451201	11529602	23059204	1.166666551
9	94158415	80707214	161414428	1.16666665
10	6,59E+08	5,65E+08	1,13E+09	1.166666664

Note S2 – Consequences of open-circuits in twin-wire resistive networks

Thin metal resistors can be damaged or even open circuited (excessive strains, scratches, contact with aggressive solutions,…). An important characteristic of thin metal resistive networks can then be the possibility to measure, in presence of an open-circuited resistor, many or, possibly, all the functional resistors (*i.e.* with no or minor damage, so that their resistance can still be measured). For instance, trifurcation is more robust than bifurcation against accidental open-circuits. In case of bifurcation, if, by any reason, a resistor is open-circuited, it will also become impossible to measure the undamaged resistors which, in absence of the open-circuited resistor, turn into series-connected. For instance, with reference to the device shown in Fig. S3a (M = 2), if a peripheral resistor (R_C in Fig. S3b) is open-circuited, two resistors (R_B and R_A in Fig. S3b) become connected in series and, therefore, may not be individually measured, so that only 2 among the 4 residual resistors can still be accurately measured. Similarly, if the central resistor is open-circuited (R_A in Fig. S3c), no resistor among the 4 residual resistors can be individually measured as each resistor is connected in series with another resistor. By contrast, if a single resistor is open-circuited, trifurcation still allows to independently measure all the residual undamaged resistors. For instance, with reference to the device shown in Fig. S3d (M = 3), if a peripheral resistor (R_C in Fig. S3e) is open-circuited, for each undamaged resistor it is still possible to determine two force paths and two sense paths, so that all the 6 residual resistors can still be individually measured. Similarly, if the central resistor is open-circuited (R_A in Fig. S3f), all the 6 residual resistors can be individually measured.

Note S3 – Estimation of the pad internal parasitic resistance

The order of magnitude of the worst case pad internal resistance can be roughly estimated as

$$R_{PAD,WC} = \rho_m \frac{X_L}{tX_W} \tag{9}$$

where X_L, t and X_W are the length, thickness and width, respectively, of a hypothetical resistor (Figure 2c) and ρ_m is the electrical resistivity of the thin metal film.

In case of deposited gold thin films with thicknesses and pad lateral dimensions in the order of nanometers and millimeters, respectively, it may be convenient to rewrite

$$\begin{aligned} R_{PAR,WC} &= \rho \frac{X_L}{tX_W} = \rho_{Au} \frac{X_{L\_mm} * 1mm}{X_{t\_nm} * 1nm * X_{W\_mm} * 1mm} \simeq \\ &= 2.44 * 10^{-8} \Omega \frac{m}{nm} \frac{X_{L\_mm}}{X_{t\_nm} X_{W\_mm}} \simeq \\ &\simeq 24\Omega \frac{X_{L\_mm}}{X_{t\_nm} X_{W\_mm}} \end{aligned} \tag{10}$$

In our test devices the thickness of gold was 100 nm (*i.e.* $X_{t\_nm} = 100$) so that

$$R_{PAR,WC} \simeq 24\Omega \frac{X_{L\_mm}}{100 X_{W\_mm}} = 0.24\Omega \frac{X_{L\_mm}}{X_{W\_mm}} \tag{11}$$

which, with similar values for X_L and X_W, is around 0.2 Ω (more accurate estimations require FEM calculations). Clearly, if necessary, R_{PAR} can be reduced by increasing the metal thickness (*e.g.* 200 nm) or the ratio X_W/X_L.

Note S4 – Validation experiments (repeated placements-removals and 180° rotations)

The mean value, maximum resistance variations and relative resistance changes during 8 repeated placements-removals were around (35.6 Ω, ± 54 mΩ, ± 0.15 %), (19.6 Ω, ± 120 mΩ, ± 0.6 %) and (21.3 Ω, ± 150 mΩ, ± 0.7 %) for R_A, $R_B + R_C$, and $R_D + R_E$, respectively. These small changes can be attributed to several non-idealities. The expected relative resistance changes of gold resistors for a ± 0.5 K temperature difference is in the order of ± 0.12 % (the temperature coefficient of thin gold metal resistances may be around 2500 ppm/K). Moreover, voltage errors introduced by the input offset voltage of the instrumentation amplifier or by spurious Seebeck voltages also introduce resistance errors (*e.g.* a ± 50 µV voltage error, with 1 mA reading current, translates into a ± 50 mΩ resistance error). However, clearly, the variations for R_A are smaller than for both $R_B + R_C$ and $R_D + R_E$. In fact, R_A is, in practice, measured by using one distinct wire for each pad or, equivalently, with a conventional method (1-wire-per-pad). By contrast, the measurements of both $R_B + R_C$ and $R_D + R_E$ require the twin-wire strategy and, therefore, are affected by the parasitic pad resistances R_{PAD}. Though the initial values of R_{PAD} are very small (*e.g.* in the order of 0.12 Ω for our devices, 100 nm) and are anyway associated to the metal film to be characterized, R_{PAD} is different for different pads, and, even for a given pad, at different iterations, can change due to slight misplacements and, more importantly, to eventual damages which can even indefinitely increase (up to open circuit) R_{PAD}. As a consequence, these experiments, besides confirming that the anti-adhesion layers effectively protect the device (otherwise all the resistances would increase), also allow to conclude that the pads are not significantly damaged by the measurement system, even after many connections and disconnections, because significant damages in a certain pad would immediately translate into irreversible and large increases of the correspondent ($R_B + R_C$ or $R_D + R_E$, depending on the damaged pad) measurements.

Note S5 – Measurements of 29R twin-wire network

The system for measuring the zero-interconnects twin-wire 29-resistors network (Figure 3a-c, Fig. S9) sequentially measures all the 29 resistances and, in order to simplify the synchronization of the multimeter data acquisition with the control PCB, also includes, as a mark, an additional, easily recognizable, measurement (*i.e.* many resistors in series, so that the measured resistance is larger than all the other measurements).

The complete data supporting this study, including all the files needed for asking a PCB company to replicate the hardware, the software and the raw data required to reproduce these findings are available to download from https://data.mendeley.com/datasets/pywhr745ns/1 (DOI: 10.17632/pywhr745ns.1).

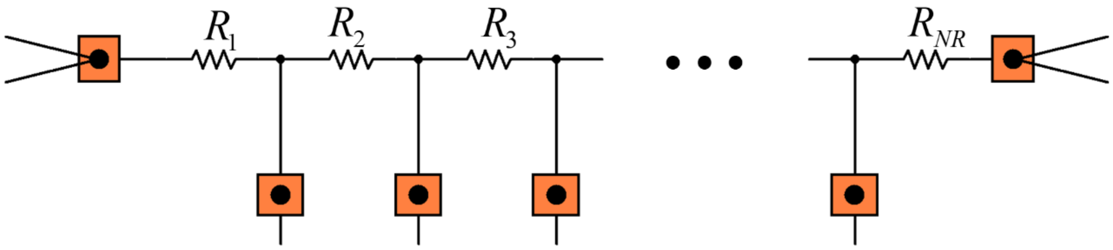

Figure S1. Network of N_R resistors with N_R + 1 pads and N_R + 3 wires.

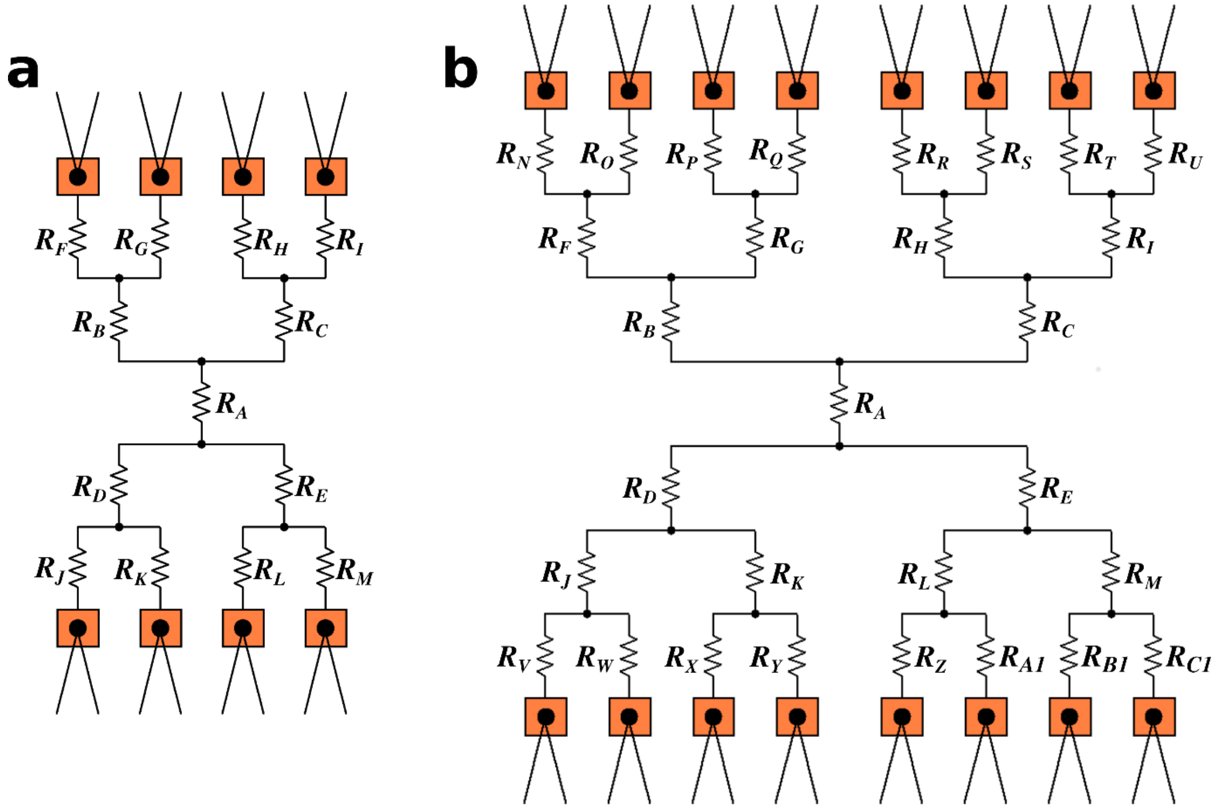

Figure S2. Twin-wire networks generated by bifurcation. (a-b) Twin-wire networks generated starting with a single initial resistor by applying two (a) bifurcation (M = 2) expansions, resulting in (N_R, N_P, N_W) = (13, 8, 16), or three (b) bifurcation expansions, resulting in (N_R, N_P, N_W) = (29, 16, 32).

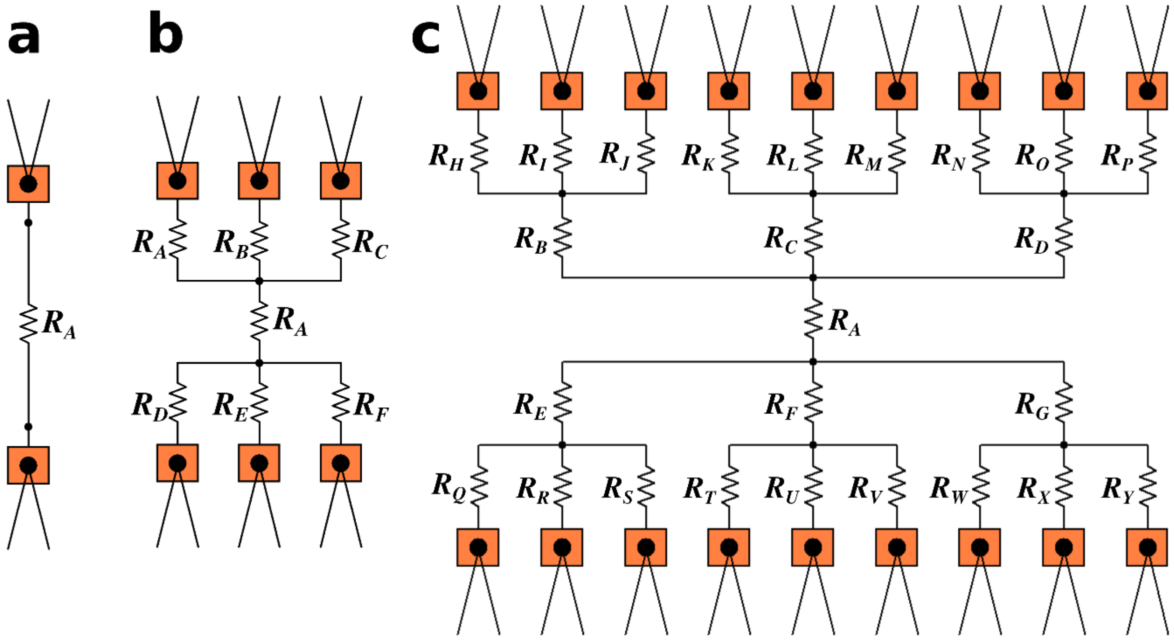

Figure S3. Twin-wire networks generated by trifurcation. (a-c) Twin-wire networks generated starting with a single initial resistor (a) by applying one (b) trifurcation (M = 3) expansions, resulting in (N_R, N_P, N_W) = (7, 6, 12) or two (c) trifurcation expansions, resulting in (N_R, N_P, N_W) = (25, 18, 36).

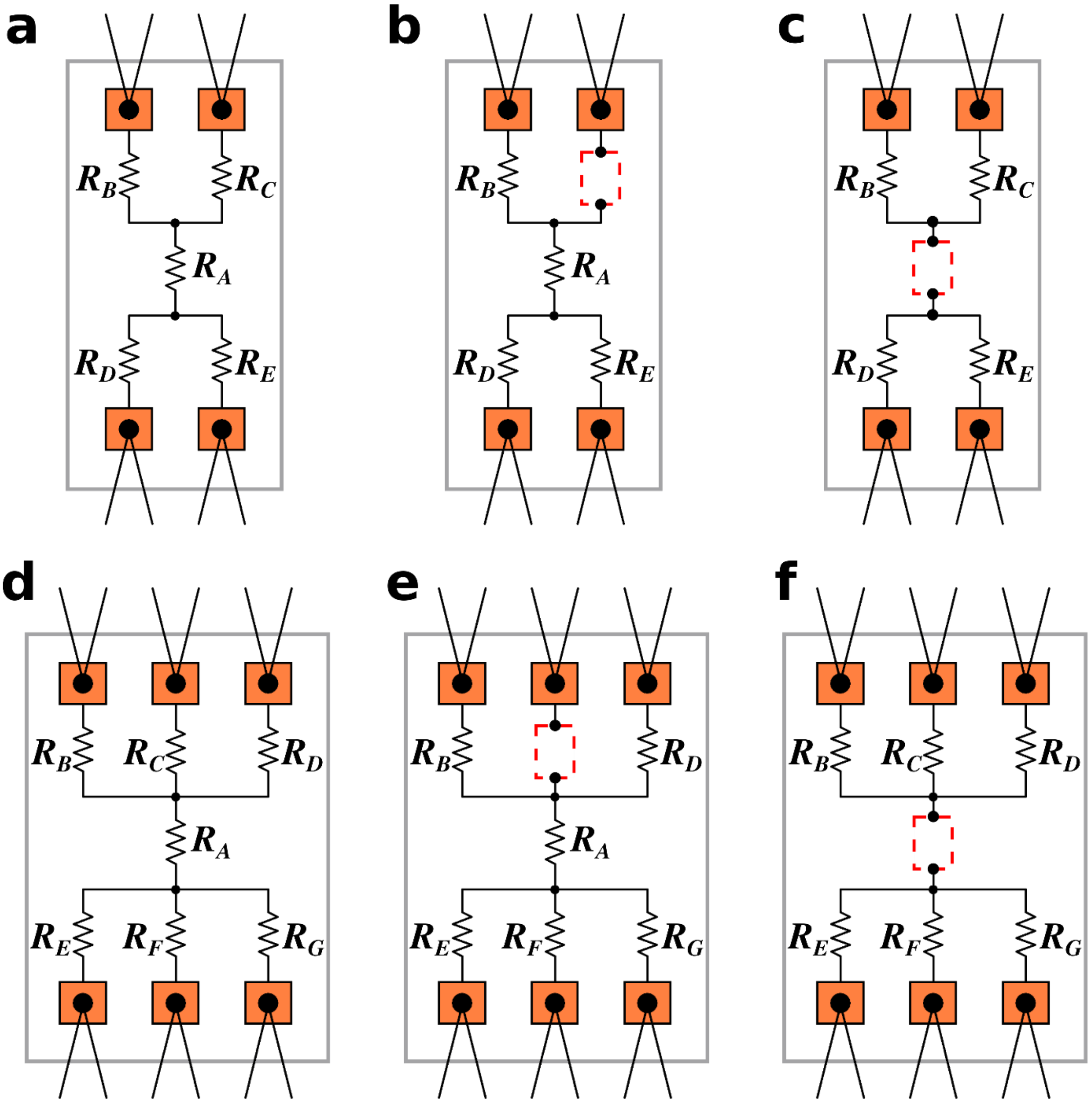

Figure S4. Effects of open-circuits on twin-wire networks. (a-c) 5 resistors obtained by applying a bifurcation expansion to a single initial resistor before any damage (a) and after a peripheral resistor (b) or a central resistor (c) is open-circuited. (d-f) 7 resistors obtained by applying a trifurcation expansion to a single initial resistor before any damage (d) and after a peripheral resistor (e) or a central resistor (f) is open-circuited.

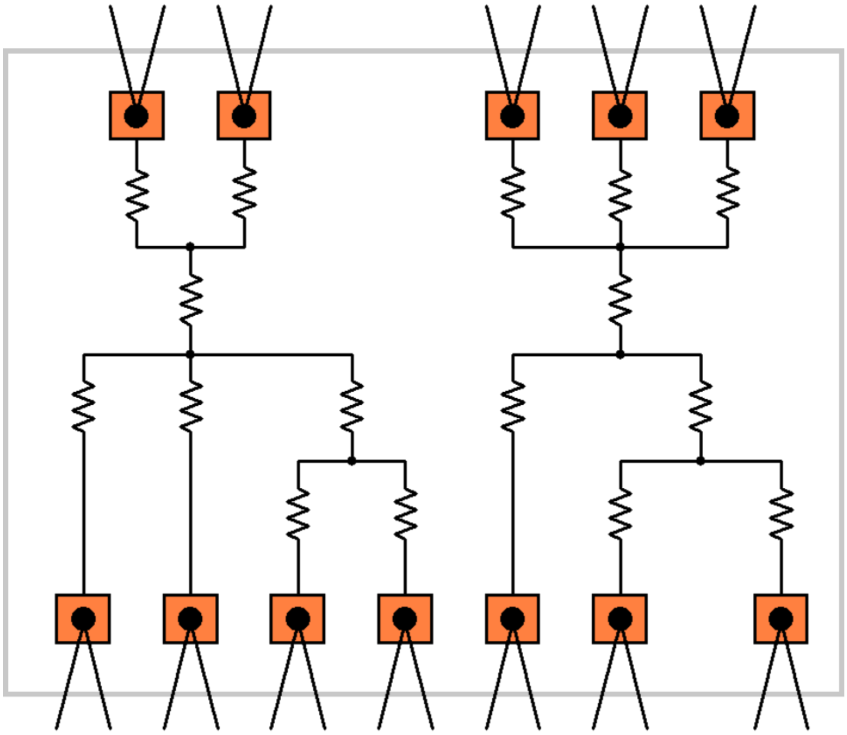

Figure S5. Twin-wire networks generated by arbitrary expansion steps. Twin-wire network generated by adding to 2 initial resistors, at every iterative step, to every free terminal a number of additional resistors equal to zero or larger than 2 (as a single added resistor would be in series and then indistinguishable).

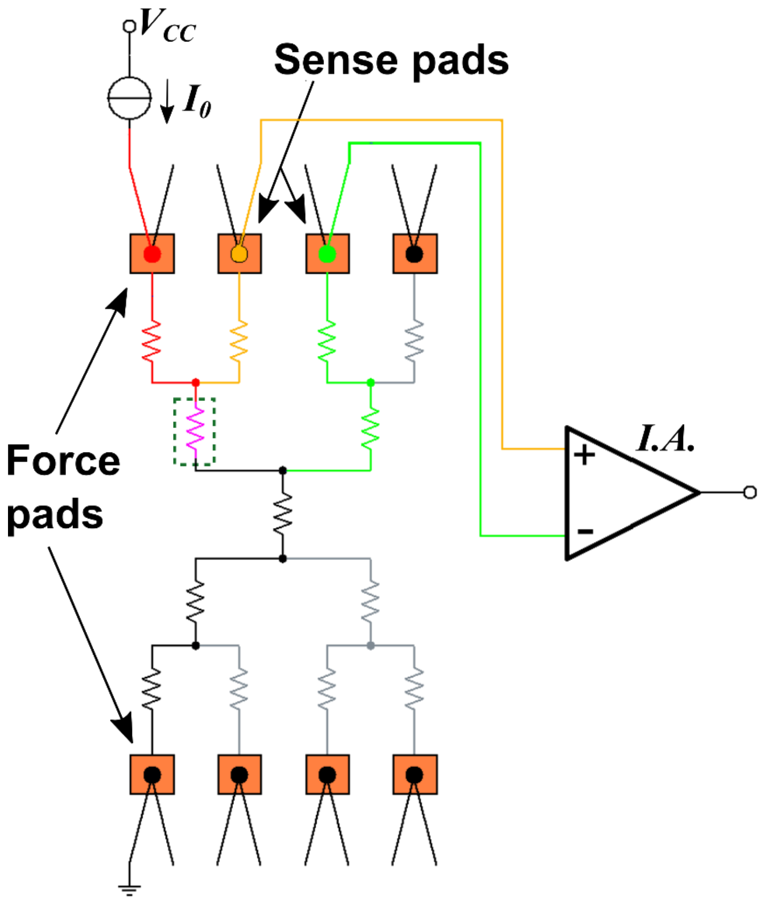

Figure S6. Measurability of each resistor in the twin-wire network. Schematic illustration that every resistor can be individually measured with a single measurement as each resistor (*e.g.* the resistor in the green dashed box in the figure) can be connected to the external instrumentation by two force pads and two sense pads. As evident, there may be more options for the connection (*i.e.* other wires can be used for both injecting the currents and sensing the voltages).

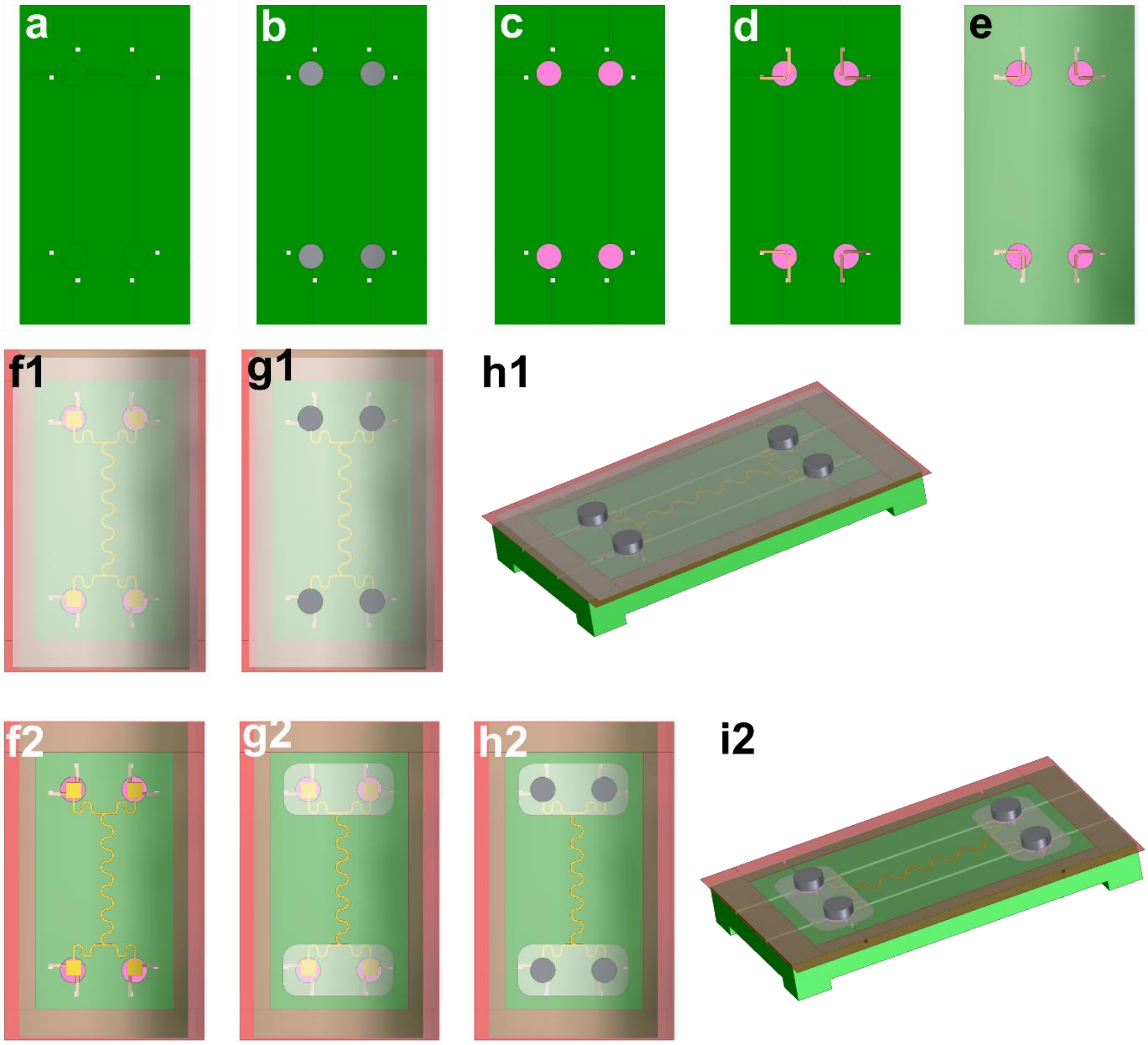

Figure S7. Schematic illustration of interconnecting systems for twin-wire networks. (a-h1), (a-i2), Schematic top views of the interconnecting system, in case of devices with (a-h1) or without (a-i2) a substrate, after each fabrication step, namely 3D-printing of the holder (a), inclusion of the internal magnets in the cavities of the 3D-printed holder (b), insulation of the internal magnets (c), insertion of the flexible interconnects in their predetermined 3D paths (d), placement of an insulating non-adhesive layer (e), placement of the device with (f1) or without (f2) a substrate, of the spacer (g2) in case there is no substrate and of magnets (g1, h2). 3D schematic representation of the final system for devices with (h1) or without (i2) a substrate.

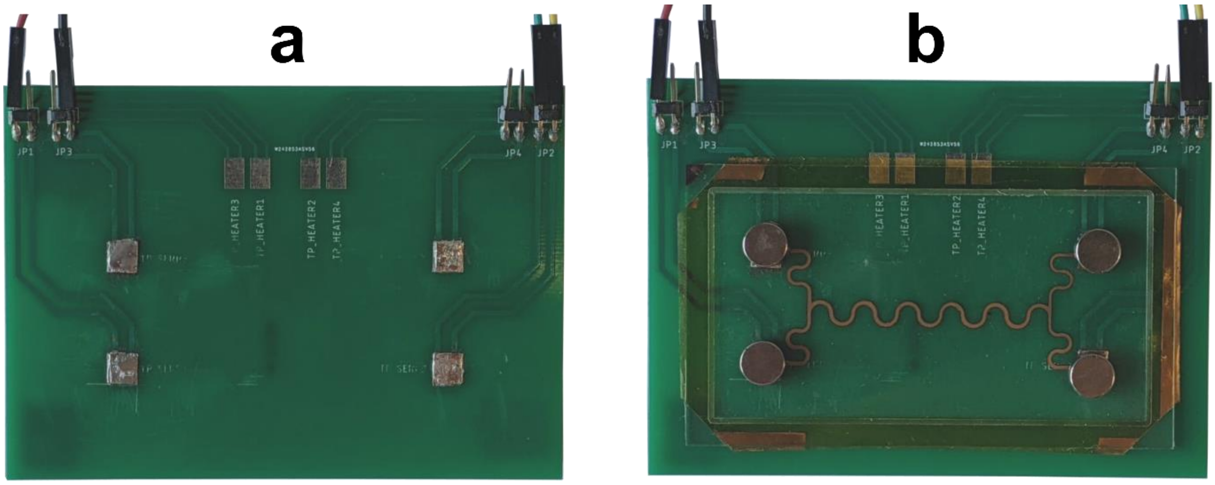

Figure S8. PCB holder for the electrical characterization of twin-wire networks. (a) Top view of the PCB holder with visible pads, copper tracks and connectors (magnets are attached on the bottom side of the PCB, in correspondence of the pads). (b) Top view of 5 thin gold resistors on PDMS, not released from the PMMA substrate, placed on the PCB holder and kept in place by additional magnets, on top of the PMMA substrate, which are auto-aligned with the magnets attached to the bottom side of the PCB.

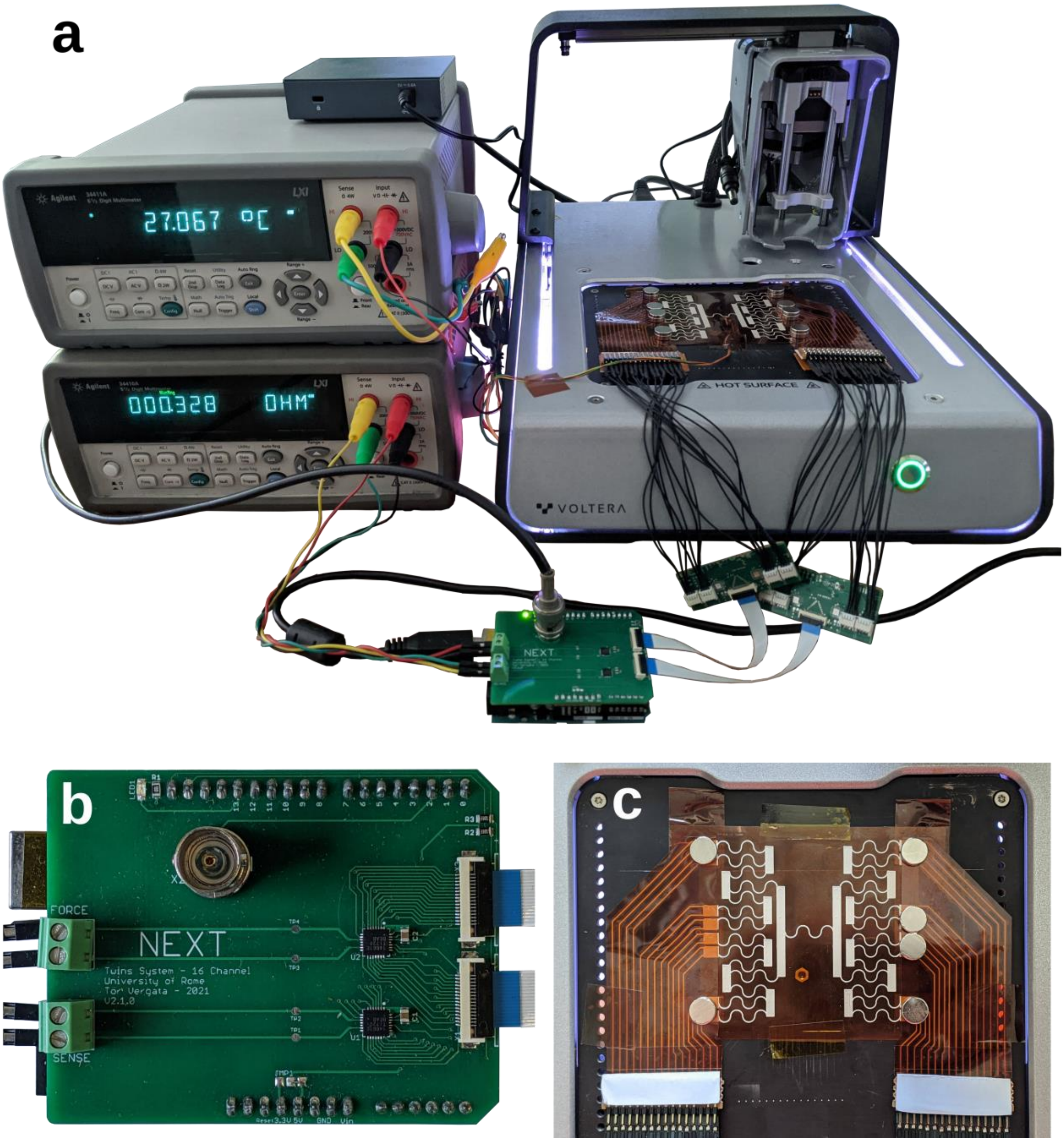

Figure S9. System for real-time measurement of 29 resistances. (a) Complete system comprising an Arduino shield, a 29 resistors device, a multimeter for performing the measurements and temporarily storing the data and a second multimeter for measuring temperature by means of a Pt100 sensor. (b) Arduino shield with 2 MAX14661 arrays of switches and a BNC connector for synchronizing the multimeter acquisition with the microcontroller driving the switches. (c) 29 resistors device printed on polyimide with magnets for connecting to the PCB control board.

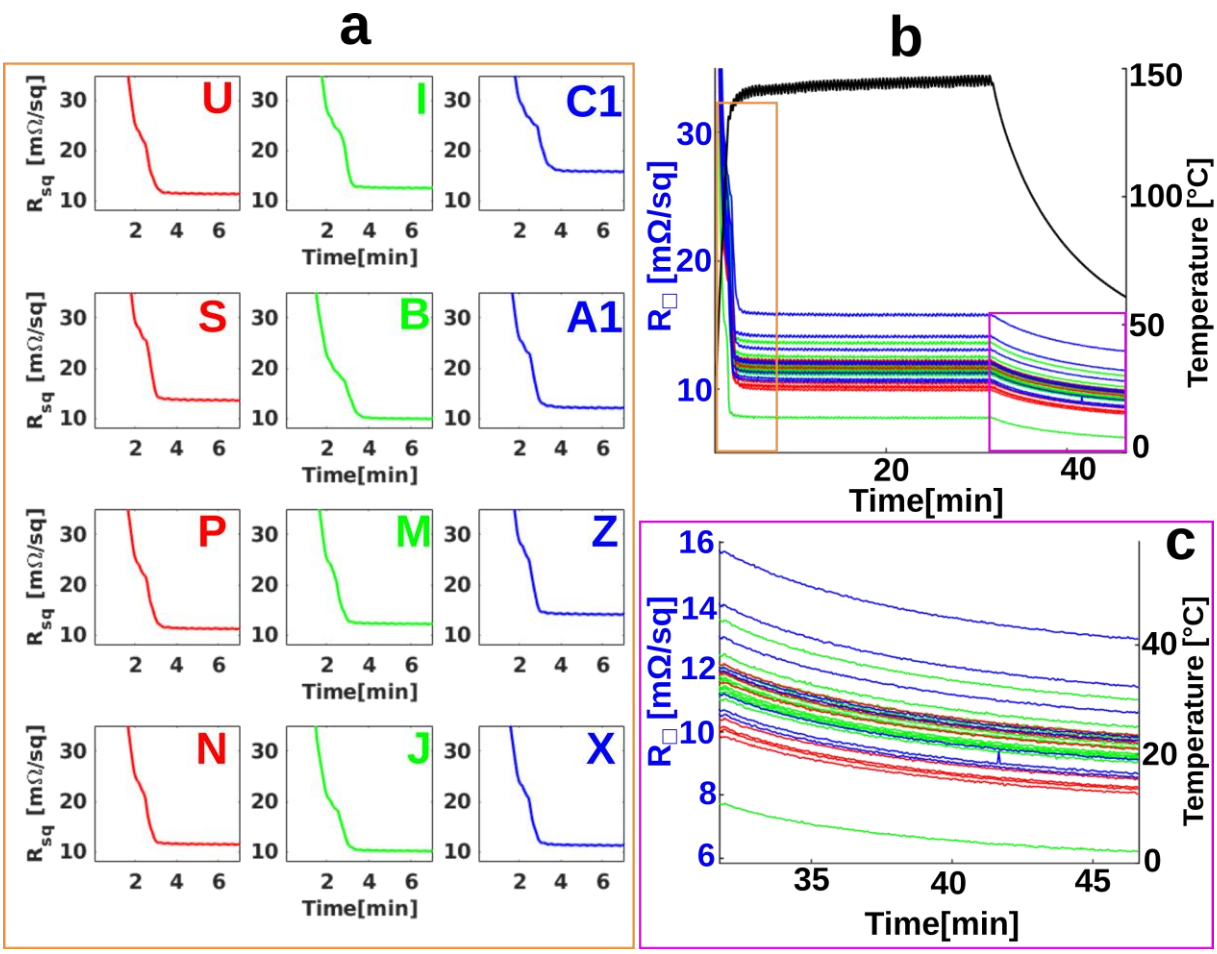

Figure S10. Sheet resistances of the twin-wire 29R silver-conductive-ink network printed on polyimide during high temperature sintering. (a) Sheet resistances of 12 representative elements of the twin-wire 29R silver-conductive-ink network during the initial period. (b) Temperature (black) and sheet resistances of the 29 elements of the twin-wire network during the entire sintering procedure. (c) Sheet resistances of the 29 elements of the twin-wire network during the final (cooling) period.

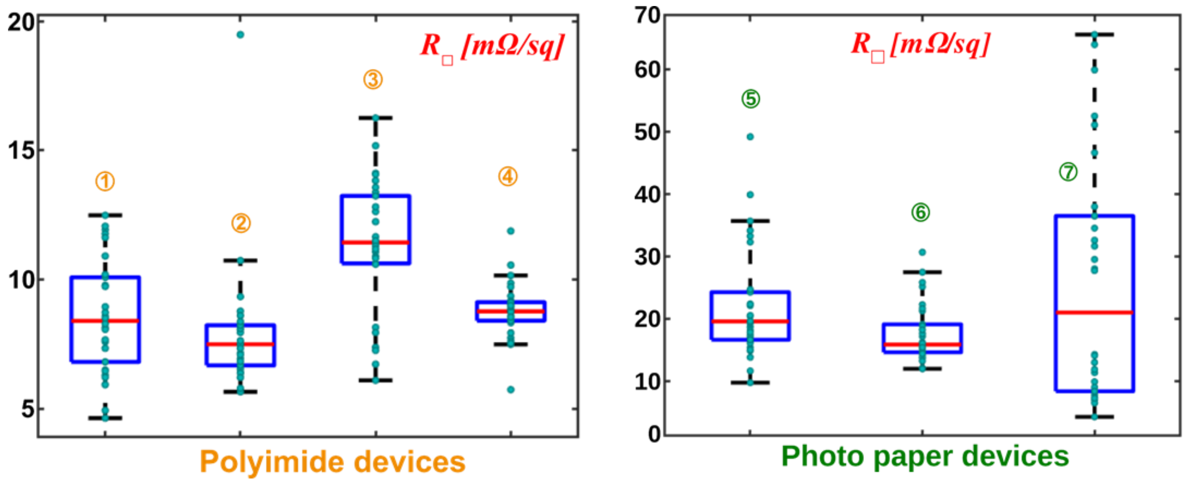

Figure S11. Sheet resistances of the 29 resistors for devices printed on polyimide (left) and on photo paper (right).

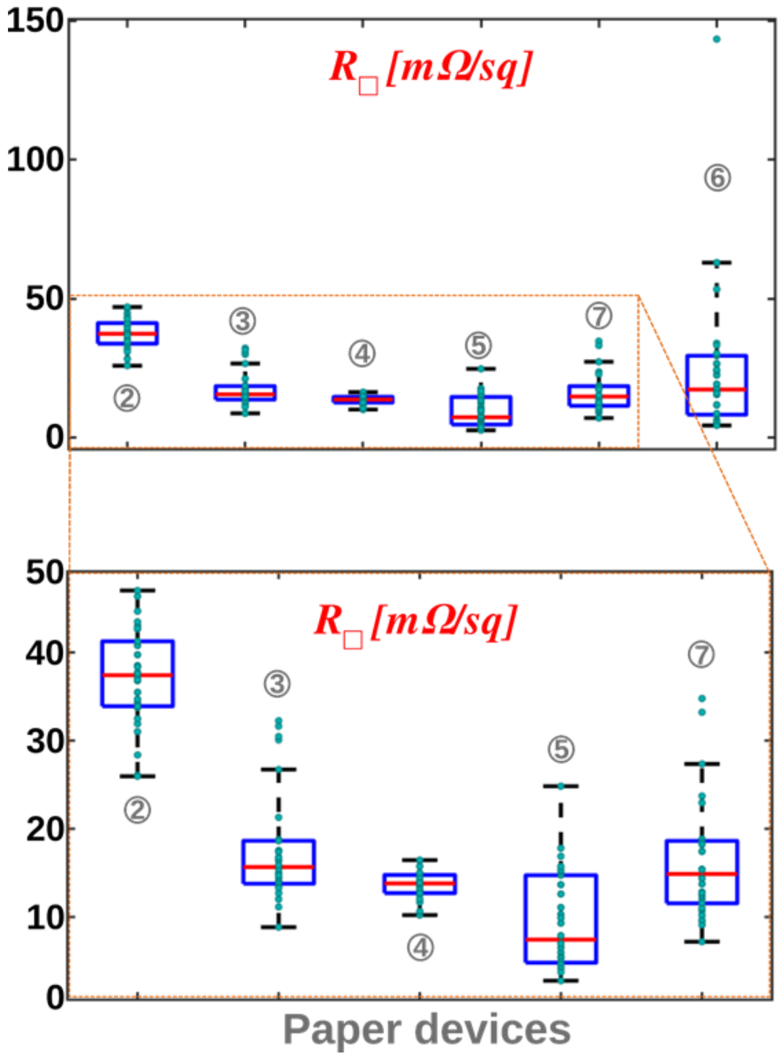

Figure S12. Sheet resistances of the 29 resistors for devices printed on paper. Sheet resistances of the 29 resistors for devices printed on paper (top), with zoom (bottom) showing the 29 sheet resistances for all devices except a device which had one resistor with an unusually high sheet resistance, almost equal to 150 mΩ/□.

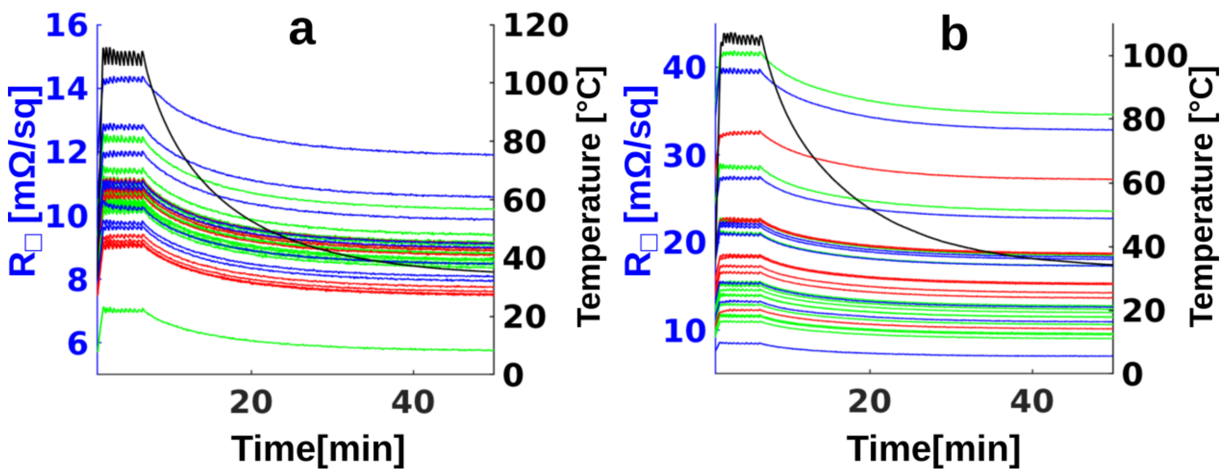

Figure S13. Temperature calibration for twin-wire 29R networks of silver conductive ink printed on polyimide and on paper. (a-b) Temperature (black) and sheet resistances of the 29 conducting elements of the twin-wire network printed on polyimide (a) and of the twin-wire network printed on paper (b).

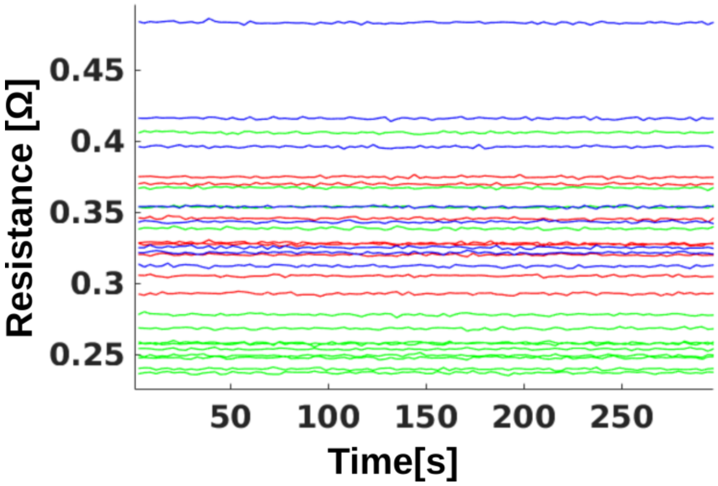

Kapton - SilverNP - Device #4			
Name	Value [Ω]	Name	Value [Ω]
R_A	0.238	R_P	0.321
R_B	0.340	R_Q	0.371
R_C	0.368	R_R	0.376
R_D	0.407	R_S	0.348
R_E	0.355	R_T	0.328
R_F	0.251	R_U	0.331
R_G	0.241	R_V	0.486
R_H	0.255	R_W	0.398
R_I	0.270	R_X	0.345
R_J	0.280	R_Y	0.417
R_K	0.259	R_Z	0.355
R_L	0.249	R_{A1}	0.327
R_M	0.260	R_{B1}	0.314
R_N	0.294	R_{C1}	0.323
R_O	0.306		

Figure S14. Resistances of a twin-wire 29R network printed on polyimide measured at room temperature. Time evolution of the 29 resistances (left) and table (right) showing the mean values of the 29 resistances (for the nomenclature of the 29 resistors please see Figure 3a or Figure S2b).

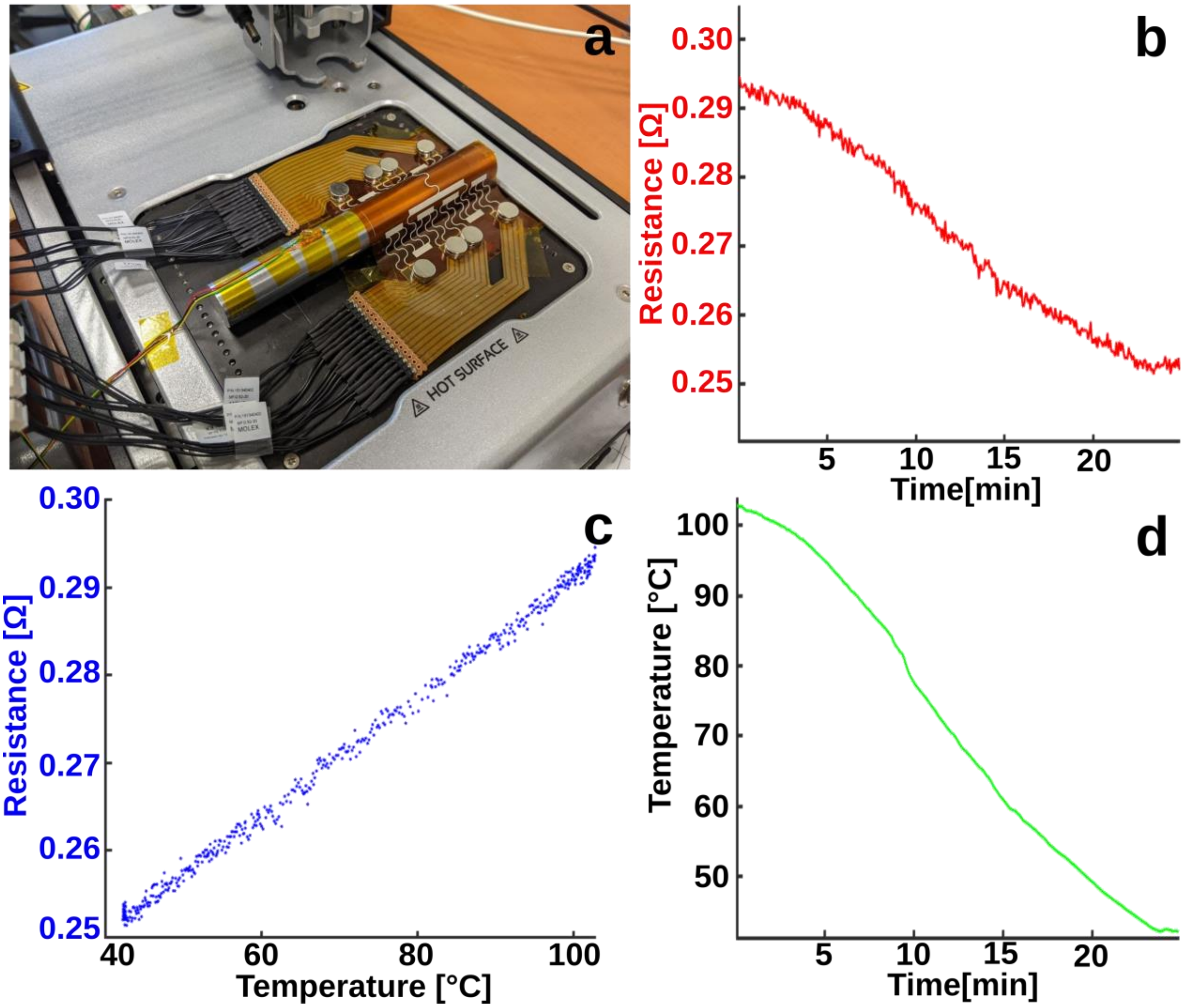

Figure S15. Characterization of resistances in a twin-wire network under deformation. (a) Photo of a twin-wire 29R network made of silver nanoparticles conductive ink printed on a flexible polyimide substrate, with the device partially attached to an aluminum cylinder (1 cm radius) placed on a temperature-controlled plate; the resistances can all be measured and their variations due to the deformation are negligible because of the small gauge factor of these resistors and of the small average strain (which can be roughly estimated as the ratio between the average distance from the neutral plane, around 65 μm, and the radius of curvature, 1 cm, i.e. about 0.65 %). (b) Resistance of the central resistor (in good thermal contact with the cylinder) when the initially heated cylinder cools to room temperature. (c-d) Resistance (c) of the central resistor versus the temperature ($\alpha\,(T_{REF}) \approx 2778$ ppm/K) of the aluminum cylinder (d) measured by a Pt100 during the cylinder cooling.
